# Supplementary material for: Nanocrystallization of Anthocyanin Extract from Red-Fleshed Apple ′QN-5′ Improved Its Antioxidant Effect through Enhanced Stability and Activity under Stressful Conditions
Source: Molecules. 2019 Apr 11;24(7):1421. doi: 10.3390/molecules24071421 (PMC6479717; doi:10.3390/molecules24071421)
Supplement: Supplementary file 1 [file molecules-24-01421-s001.pdf]

Supplemenatry Materials

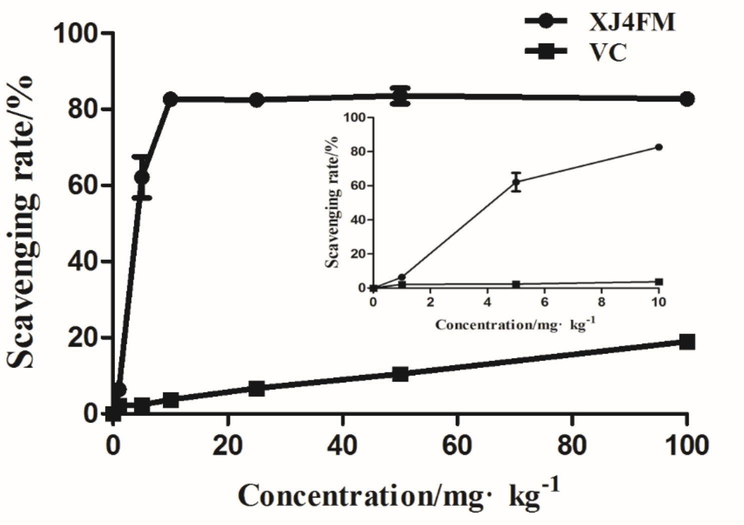

Figure 1. The relationship between DPPH radical scavenging rate and concentration of anthocyanin in red-fleshed apple 'QN-5'.
